# Parental support of the Canadian 24-hour movement guidelines for children and youth: prevalence and correlates

**DOI:** 10.1186/s12889-019-7744-7

**Published:** 2019-10-28

**Authors:** Ryan E. Rhodes, John C. Spence, Tanya Berry, Guy Faulkner, Amy E. Latimer-Cheung, Norman O’Reilly, Mark S. Tremblay, Leigh Vanderloo

**Affiliations:** 1 Behavioural Medicine Laboratory, School of Exercise Science, Physical and Health Education, University of Victoria, PO Box 1700 STN CSC, Victoria, BC V8W 2Y2 Canada; 2 University of Alberta, Edmonton, AB Canada; 3 University of British Columbia, Vancouver, BC Canada; 4 Queen’s University, Kingston, ON Canada; 5 University of Guelph, Guelph, ON Canada; 6 Children’s Hospital of Eastern Ontario Research Institute, Ottawa, ON Canada; 7 ParticipACTION, Toronto, ON Canada

**Keywords:** Theory of planned behavior, Perceived behavioral control, Intention, Attitude, Parent-child relationship, Parenting

## Abstract

**Background:**

To explore the prevalence of parental support for meeting the Canadian 24-Hour Movement Guidelines for Children and Youth, identify key interactive support profiles among the four movement behaviors, and investigate subsequent sociodemographic and social cognitive correlates of these profiles.

**Methods:**

A sample of Canadian parents (*N* = 1208) with children aged 5 to 17 years completed measures of the theory of planned behavior (TPB), and support of the four child movement behaviors via questionnaire. Differences in the proportion of parents supporting these four health behaviors were explored and demographic and social cognitive (attitude and perceived control) correlates of combinations of parental support for the four health behaviors were evaluated.

**Results:**

Child and youth sleep behavior had the highest parental support (73%) and moderate to vigorous physical activity support had the lowest prevalence (23%). Interactive profiles of the four movement behaviors yielded six primary clusters and comprised wide variation from parents who supported none of these behaviors (19%), to parents who supported all four behaviors (14%). These profiles could be distinguished by the age of the child (younger children had higher support) and the gender of the parent (mothers provided more support), as well as constructs of the TPB, but TPB cognitions were more specific predictors of each health behavior rather than general predictors of aggregate health behavior clusters.

**Conclusions:**

Teenagers and fathers may represent key targets for parental support intervention of the 24-Hour Movement Guidelines. Intervention content may need to comprise the underlying foundations of attitude and perceived behavioral control to change parental support while considering the unique features of each health behavior to maximize related intervention effectiveness.

## Background

The health benefits of regular physical activity for children and youth are indisputable [1, 2] and a low level of sedentary screen time [3] and assurance of adequate sleep [4] are also associated with desirable health indicators and outcomes. Furthermore, these movement behaviors interact, demonstrating that the composition of behaviors across the whole day are associated with health outcomes [5, 6]. In response to this evidence, 24-h movement guidelines were developed to provide integrated recommendations for physical activity, sedentary behavior, and sleep for Canadian children and youth [7]. Of concern, less than 20% of Canadian children aged 5–17 years adhere to these healthy movement behavior guidelines [8, 9]. Thus, the promotion of healthy movement behaviors during childhood is important for public health, and understanding the factors that can facilitate effective interventions is of critical importance.

Multiple factors are associated with child and youth movement behaviors spanning from individual-level dispositions to the various settings where children live, study, and play [10–14]. In particular, parents and the household setting can play important roles in the health behavior of children. For instance, parents represent critical agents in bedtime routines for sleep [15] and screen-time access and duration [16] as well as being facilitators of physical activity during family time [17, 18]. Because of this clear link between parental support and child and youth health behavior, parental support of health behavior is being studied as a critical behavior unto itself [19–22].

Parental support is an overarching term used to represent the interactions between a parent and their children in promoting health behavior [23, 24]. We used the components of logistical support (e.g., facilitating the health behavior, transportation to activities), encouragement (e.g., providing information and praise about the health behavior, spectating), and co-activity (i.e., parent-facilitated support via activity together and not mere modeling) previously proposed for physical activity [25]. In addition, we included the component of regulatory support (e.g., enforcing rules, setting limits) for sleep and screen time restriction [24]. At present, the best evidence for physical activity parenting support aligns with these logistical, encouragement, and co-activity support components [23, 26–30], while sleep support and screen time reduction are also correlated with regulatory support [15, 16, 24]. However, the prevalence of parental support for meeting child and youth movement guidelines is not well understood. Indeed, we were not able to locate any studies that provided an estimate of support prevalence among parents for child movement behaviors at the level of recommended guidelines. One recent study did provide estimates of the prevalence of parental support for physical activity and restricting screen time but the frames of the questions were generalized (e.g., encourage my child to walk or cycle to places) and not anchored to guidelines [24]. Understanding the prevalence of parents who are in line with the recommended guidelines is helpful because it provides information on which health behaviors are being supported the most among parents and gives direction for where interventions may be most (and least) needed when targeting parental support.

Thus, the first purpose of this paper was to examine the prevalence of parental support for meeting the Canadian 24-Hour Movement Guidelines for Children and Youth [7]. We hypothesized that sleep behavior was likely to have the highest support among parents. This was based on the rationale that the benefits of parental support of sleep are well-established [31], the behavior is highly routinized, the support behavior is low burden (e.g., short in duration each night, no financial cost), and that parents are most likely available for the opportunity to provide sleep support (i.e., home in the evening). Of course, the purpose of the 24-Hour Movement Guidelines for Children and Youth is to recognize and appreciate the interactivity of sleep, light physical activity (LPA), moderate-vigorous intensity physical activity (MVPA), and sedentary behavior on health outcomes [7]. Therefore, a secondary purpose was to explore the interactive profiles of parental support across these four health behaviors. Given the novelty of this study, we had no specific a priori hypotheses about which of the 16 behavior combination profiles would emerge to describe parents but we did not expect these profiles to be equally distributed.

The third purpose was to explore the sociodemographic and social cognitive correlates of these parental support profiles of the 24-Hour Movement Guidelines for Children and Youth. Previous research on sociodemographic correlates has produced mixed and generally null results when exploring factors such as social capital, gender of parent, age of parent, parental ethnicity, and family income, yet child age has been a fairly consistent correlate of parental support [24, 25, 32–36]. Specifically, younger children receive more parental support than older children and youth. Similar findings have been reported for sleep [31] and sedentary behavior [16], and likely reflect the increasing autonomy of behavior granted by parents as their children move through adolescence. Based on this evidence, we hypothesized that child age would correlate with the interactive profiles of parental support of the 24-Hour Movement Guidelines. Specifically, we hypothesized that parents of younger children would be present in the higher support clusters (i.e., support of more behaviors) than the lower support clusters compared to parents of adolescents.

To explore the social cognitive correlates of the interactive profiles of parental support, we employed key antecedent constructs of the Theory of Planned Behavior (TPB) [37]. This theory suggests that the foundations of behavioral motivation and subsequent action are predicated on affective (enjoyment) and instrumental (utility) attitudes, subjective norm (perceived social pressure), and perceived behavioral control (ease/difficulty of performing the behavior). Perceived control is comprised of at least two fundamental composites relevant to parenting support: *perceived opportunity* and *perceived capability* [37, 38]. Perceived opportunity is the perceived time and available access that can allow one to perform the behavior [38]. Perceived capability represents perceptions of physical and mental ability, capacity, or competence to perform a specific circumscribed behavior [39]. The TPB has been validated as an effective framework to explain parental support of child and youth physical activity, with perceived behavioral control showing the largest association with parental support, followed by attitude [21, 22, 25, 40–42]. Thus, we hypothesized that higher values of all TPB constructs would be associated with higher support clusters (i.e., support of more behaviors) than lower support clusters compared to parents with lower values of these TPB constructs.

Finally, based on the integrated nature of the 24-Hour Movement Guidelines for Children and Youth, and the critical importance of understanding multiple health behavior change [43–45], we sought to explore the predictive compatibility of parental support social cognitions compared to their generality of prediction across other health behaviors in these integrated profiles. The TPB [37] suggests that cognitions are context and action specific (i.e., an attitude about supporting physical activity should predict physical activity support) and may not generalize to other actions or contexts (i.e., an attitude about supporting physical activity may not link to supporting sleep). Despite this tenet of specificity, there is great potential benefit in interventions if health promotion of one behavior can affect subsequent health promotion of another behavior and this is highly relevant to integrated health behavior guidelines. Thus, an exploration of the compatibility versus generality of support cognitions when predicting parental support of the 24-Hour Movement Guidelines has relevant value. We hypothesized, based on the TPB [37], that social cognitions about parental support would favor prediction compatibility over prediction generality.

## Method

### Study design and participants

This is a secondary analysis of a cross-sectional study (see [46, 47] as prior papers using these data), comprised of a national Canadian opt-in online panel survey via a hired vendor, Maru/Matchbox that was carried out in October 2017. Participants were recruited from Maru/Matchbox’s consumer online panel database of over 110,000 individuals with the sample reflecting the Canadian census in terms of age, sex, region, income, employment, and language spoken [48]. Maru/Matchbox’s panel members are typically recruited via word of mouth, referrals, campaigns, and partnering communities. For the current study, a random sample of 1208 parents with children between the ages of 5 and 17 years was selected by Maru/Matchbox. Once respondents agreed to participate, they were allowed 2 weeks to complete the online survey in either French or English. Human research ethics for this study was obtained from a co-author’s Research Ethics Board (ALC).

### Measures

All health behaviors for children and youth were defined according to the Canadian 24- Hour Movement Guidelines for Children and Youth [49]. For clarity, measures for each behavior were completed separately. Specifically, the definition of the health behavior was provided to respondents which was then followed by the definition of parental support for that behavior (see Additional file 1: Table S1). Parents were asked to think of their child whose birthday was closest to the date of the study and use that child as the referent for answering questions.

#### Demographics and health behaviors

Information on household income, education, employment status, number of children in the home, age of the child who was the target for the questions in the survey were provided by the parent. Parents also reported perceptions of their child’s MVPA, LPA, sleep, and screen time (see Additional file 2: Table S2).

#### Attitude about child support of physical activity

Two items for instrumental (e.g., harmful-beneficial, useless-useful) and affective (e.g., unenjoyable-enjoyable, unpleasant-pleasant) properties of an attitude for parental support of each child health behavior were included using seven-point semantic differential scaling [50]. Reliabilities were acceptable for MVPA support (instrumental attitude α = .89; affective attitude α = .84), LPA support (instrumental attitude α = .92; affective attitude α = .92), sleep support (instrumental attitude α = .92; affective attitude α = .92), and screen time restriction support (instrumental attitude α = .89; affective attitude α = .93).

#### Perceived behavioral control over child physical activity support

As recommended by previous research [38, 39, 51], perceived behavioral control measured specific components for perceived opportunity (1 item: I will have an opportunity to support my child’s ____) and perceived capability (2 items: I have the ability to support my child’ s ______; I am capable of supporting my child’s ___). To account for any confounds in perceived capability versus perceived willingness [39], all items were measured with a phrase that held motivation to support constant (i.e., “if I wanted to”) and featured five-point Likert scaling from (1) strongly disagree to (5) strongly agree. Reliabilities were acceptable for MVPA support (perceived capability α = .91), LPA support (perceived capability α = .94), sleep support (perceived capability α = .92), and screen time restriction support (perceived capability α = .93).

#### Parental support behavior

Participants were asked about the frequency they: (1) encourage [their] child to participate in moderate to vigorous intensity physical activity or sport; (2) play outside with [their] child or do moderate to vigorous intensity physical activity with their child; and, (3) drive or provide transportation to a place [their] child can do moderate to vigorous intensity physical activity or play sports [42]. Measurement of the other health behaviors followed similar item content. Specifically, LPA included the items “encourage your child to participate in light physical activities around the house or outdoors” and “engage in light physical activities with your child.” Sleep support included the items “encourage your child to sleep between 9-11 hours per night” and “enforce your child’s sleep schedule.” Screen time restriction support included the items “encourage your child to stop sitting and watching screens” and “enforce your child’s screen time schedule.” Responses were scored as 1 (never/rarely), 2 (1–2 times per week), 3 (3–4 times per week), 4 (most days) and 5 (daily). MVPA support (α = .75), LPA support (α = .80), sleep support (α = .83) and screen time (α = .86) restriction support all showed adequate reliability and the measure has shown predictive validity of child and youth physical activity [46].

### Analysis plan

Data were analysed in SPSS 20 (SPSS Inc., Chicago, IL, USA). Normality of all variables was checked and descriptives of all variables were computed. To create the dichotomies of parental support for the various child health behaviors, the support variables were formatted to include “most days (4)” and “every day (5)” responses as support for that guideline. By contrast, those participants whose responses were among the “no days/rarely (1)” to “3-4 days per week (3)” were scored as failing to meet support for a behavioral guideline. Though dichotomies truncate the range of a distribution, this is appropriate when assessing the Canadian 24-Hour Movement Guidelines for Children and Youth because one either meets this criterion or they do not. Furthermore, our ratio-level scale of measurement (days of the week) allowed for a strong correspondence with these guidelines. Potential differences in the proportion of parents supporting these four health behaviors were subsequently explored using Cochrane’s Q statistic.

Combinations of parental support for the four health behaviors of the Canadian 24- Hour Movement Guidelines were then created (i.e., proportions of those in each of the16 possible combinations). Demographic and social cognitive (attitude and perceived control) correlates of these combinations were evaluated using chi-square analyses and analysis of variance, respectively. Considering a small-medium effect size (*f* = .17), an alpha of .01, and a power of .80, 57 participants were needed in a particular 24-Hour Movement profile to be included in the analyses [52]. Significance was set at *p* < .01 to control for type 1 error. Given the large sample size, effect sizes were used to aid in the interpretation of the inferential statistics. Specifically, we used *n*^*2*^ = .04 as the minimum recommended effect size for the social sciences based on Ferguson’s [53] recommendations. Bonferroni post-hoc tests of mean differences were then applied to explore where the differences between the profiles may have occurred. Finally, descriptive analyses of the post-hoc tests were compiled to explore the compatibility and generality of the associations between the social cognitive constructs and the parental support combinations. Compatibility tests were considered those post-hoc tests between a social cognition for a specific behavior and its subsequent positive association with meeting the guideline for that behavior within the clusters (e.g., was affective attitude about support for MVPA higher in clusters where support for the MVPA guideline was being met compared to clusters where it was not being met?). Generality tests were considered those post-hoc tests between a social cognition for a behavior and its positive association with meeting the guideline for a different behavior or behaviors (e.g., was affective attitude about support for MVPA higher in clusters where subsequent guidelines were being met, independent of whether the MVPA guideline was being met?).

## Results

### Participant characteristics

Congruent with the regional stratification sampling plan, representation across Western Canada (Alberta = 10.3%; British Columbia = 14.2%; Manitoba = 3.0%; Saskatchewan = 3.6%), Central Canada (Ontario = 36.8%; Quebec = 24.2%), Atlantic Canada (New Brunswick = 2.2%; Newfoundland/Labrador = 1.7%; Nova Scotia = 3.3%; PEI = 0.5%) and the territories (North West Territories = 0.2%; Nunavut = 0.1%) was reflective of Canadian demographics [48]. Parents reported an average of 1.8 children (*SD* = 1.01) and an average child age of 11.59 years (*SD* = 3.81) when asked about the age of the child being considered in this survey. Parent respondents were 52.3% female, 52.1% had completed a University degree, 54.0% had household income above $75,000 CDN, and 69.1% were employed. These figures correspond with national patterns [54]. In terms of health behaviors of the 24-Hour Movement Guidelines, findings showed that based on parent reports, 12.7% of their children were meeting MVPA guidelines, 30.5% were meeting LPA guidelines, 25.8% were meeting screen time guidelines, and 69.8–72.2% were meeting sleep guidelines.

### Parental support of the 24-hour movement guidelines for children and youth

**Fig. 1:**
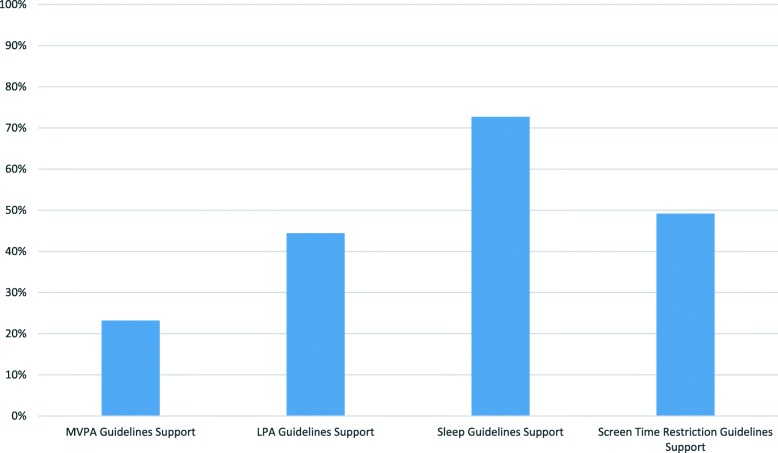
Percentages of parents regularly supporting their children in specific behaviors of the 24-Hour Movement Guidelines. Note: All behavioral support percentages significantly different from one another *p* < .01 using the Cochrane Q statistic. LPA = light intensity physical activity; MVPA = moderate to vigorous intensity physical activity

Figure 1 displays the distribution of parental support for the four health behaviors in the 24-Hour Movement Guidelines. The percentages of support on most days of the week or higher for these behaviors were significantly different from one another in terms of an omnibus test (Cochrane’s *Q* = 796.53; *p* < .01) and subsequent univariate follow-up comparisons (all tests *p* < .01). Specifically, sleep (9–11 h) had the highest support with 72.7% of parents reporting they supported this behavior on most days of the week. This was followed by regular support of screen time restriction (49.2% of parents supported) and LPA (44.4% of parents supported). Support of child and youth MVPA on most days of the week proved to have the least endorsement at 23.2%.

**Fig. 2:**
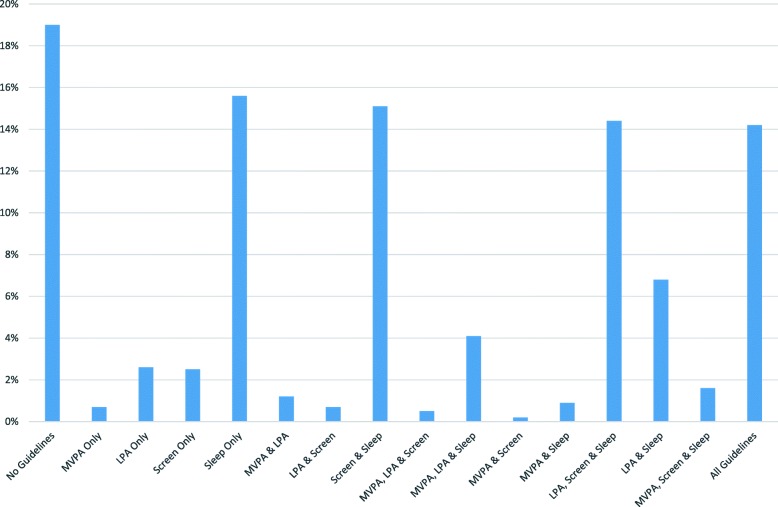
Proportions of parents regularly supporting their children in combinations of behaviors of the 24-Hour Movement Guidelines. Note: LPA = light intensity physical activity; MVPA = moderate to vigorous intensity physical activity

Because the 24-Hour Movement Guidelines for Children and Youth are considered in aggregate format for improved health outcomes [7], parental support at public health guidelines of these four behaviors were combined to explore the distribution of the 16 possible behavioral support clusters. These results can be found in Fig. 2. The profiles of these behavioral clusters yielded the following distributions: 1. no support for any guideline (19.0%; *n* = 230), 2. MVPA support only (0.7%; *n* = 8), 3. LPA support only (2.6%; *n* = 31), 4. screen time support only (2.5%; *n* = 30), 5. sleep support only (15.6%; *n* = 189), 6. MVPA & LPA support (1.2%; *n* = 14), 7. LPA & screen time support (0.7%; *n* = 9), 8. screen time and sleep support (15.1%; *n* = 183), 9. MVPA, LPA, & screen time support (0.5%; *n* = 6), 10. MVPA and screen time support (0.2%; n = 2), 11. MVPA and sleep support (0.9%; *n* = 11), 12. LPA, screen time and sleep support (14.4%; *n* = 174), 13. LPA and sleep support (6.8%; *n* = 82), 14. MVPA, LPA and sleep support (1.6%; *n* = 19), 15. MVPA, LPA and sleep support (4.1%; *n* = 49) and 16. support of all guidelines (14.2%; *n* = 171).

### Correlates of parental support clusters of the 24-hour movement guidelines for children and youth

Following our a priori power analysis of cell sizes required to perform analyses of the parental support clusters (*n* > 57), we retained six of the 16 possible clusters: 1. no support for any guideline (*n* = 230), 2. sleep support only (*n* = 189), 3. screen time and sleep support (*n* = 183), 4. LPA and sleep support (*n* = 82), 5. LPA, screen time and sleep support (*n* = 174), and 6. support of all guidelines (*n* = 171), which collectively represented 85.1% of the original sample.

Table 1 provides details of parental and child sociodemographic variables as correlates of these six parental support clusters of the 24-Hour Movement Guidelines. Parental marital status, education, household income, and occupational status were not related to these clusters (*p* > .11). By contrast, age of the child [χ^2^ (5) = 135.99; *p* < .01], sex of the parent [χ^2^ (5) = 22.74; *p* < .01], and number of children in the home [χ^2^ (5) = 11.81; *p* < .01] were associated with differences in the parental support clusters of these four health behaviors. Specifically, parents of teenagers 13+ years (67%) were more prevalent in the group of parents who reported no regular support of any guidelines compared to any of the other clusters where the support of a guideline was present (*p* < .01). In turn, parents supporting sleep guidelines only were more likely (*p* < .01) to be the parents of teenagers (42.9%) than the clusters of parents who supported multiple guidelines (29.8 to 18.4%). Fathers also represented a higher proportion in the cluster of parents who reported no support for any of the 24-Hour Movement Guidelines (59.6%) when compared to clusters where the support of a guideline was present (*p* < .01). A higher proportion of fathers were also present (*p* < .01) in the support of sleep and screen time cluster (47.5%) compared to the LPA, screen time, and sleep cluster (38.5%) and the support of all four guidelines cluster (37.8%). Finally, those parents with three or more children/youth in the household were more likely (*p* < .01) to reside in the support for screen time and sleep cluster (19.7%) compared to those who reported no support for any guideline (10.6%).

**Table 1:** Demographic correlates of behavioral clusters of parental support of the 24-Hour Movement Guidelines for Children and Youth

Variable	(1) Not supporting any guideline (*n* = 230)	(2) Supporting sleep only (*n* = 189)	(3) Supporting screen time and sleep (*n* = 374)	(4) Supporting LPA and sleep (*n* = 84)	(5) Supporting LPA, Sleep, and screen time (*n* = 174)	(6) Supporting all guidelines (*n* = 171)	*Χ* ^2^_5_	*p*	Post-Hoc
Age									
% < 13 years	33.0	57.1	75.4	70.2	81.6	73.2	136.0	.00	1 < 2 < All
Sex of Parent									
% female	40.4	55.0	52.5	53.8	61.5	62.2	22.7	.00	1 < All; 3 < 5,6
Number of Children									
% < 3	89.4	88.8	80.3	81.2	82.5	81.7	11.8	.04	3 < 1
Marital status									
% Married/	78.3	82.0	85.2	87.1	86.8	86.6	8.92	.11	NA
Common-law					54.0	54.9	3.16	.68	NA
Education									
% University	49.6	47.6	50.8	55.0					
Degree									
Income									
% > $75,000	51.3	58.7	55.2	58.5	53.4	56.1	3.35	.65	NA
Occupational Status	70.0	67.7	71.0	70.8	68.4	62.2	2.97	.71	NA
% Employed									

Note: *LPA* Light physical activity. Post Hoc tests are Bonferoni corrected to *p* < .01. *NA* Not applicable

Table 2 details the associations of social cognitive constructs (attitudes and control over support for child/youth MVPA, LPA, sleep, and screen time) with the six parental support clusters of the Canadian 24-Hour Movement Guidelines. All social cognitive constructs significantly differentiated these parental support clusters (*p* < .01). The size of these effects, however, showed reliable differences among the behaviors. Specifically, social cognitions about MVPA support of the Canadian 24-Hour Movement Guidelines for Children and Youth was consistently within the medium effect size range (η^2^ = 0.09 to 0.11) when differentiating among the six clusters [55]. LPA support cognitions were consistently in the low-end of the large effect size range (η^2^ = 0.15 to 0.18), followed by sleep support cognitions (η^2^ = 0.16 to 0.22). By contrast, screen support social cognitions were the highest reported effect sizes across all four behaviors when differentiating among the six parental support clusters (η^2^ = 0.20 to 0.29).

**Table 2:** Social cognitive correlates of behavioral clusters of parental support of the 24-Hour Movement Guidelines for Children and Youth

Variable	(1) Not supporting any guideline (*n* = 230)	(2) Supporting sleep only (*n* = 189)	(3) Supporting screen time and sleep (*n* = 374)	(4) Supporting light PA and sleep (*n* = 84)	(5) Supporting light PA, Sleep, and Screen time (*n* = 174)	(6) Supporting all guidelines (*n* = 171)	*F*_51023_	η2	Post-Hoc
Affective Attitude									
MVPA support	4.81 (1.27)	5.13 (1.17)	5.40 (1.40)	5.42 (1.11)	5.56 (1.00)	6.00 (0.89) 6.18	26.23	0.11	1 < All; 2 < 5 < 6; 3,4 < 6
LPA support	4.83 (1.24)	5.40 (0.94)	5.54 (0.97)	5.73 (1.03)	5.94 (0.82)	(0.88)	44.1437.94	0.180.16	1 < ALL; 2,3 < 5,6; 4 < 6
Sleep support	4.51 (1.26)	5.24 (1.27)	5.51 (1.23)	5.38 (1.30)	5.80 (1.09)	5.98 (1.06)	49.64	0.20	1 < All; 2 < 5,6; 3,4 < 6
Screen support	3.64 (1.49)	3.65 (1.49)	4.70 (1.53)	3.90 (1.49)	4.82 (1.54)	5.56 (1.28)			1,2,4 < 3,5 < 6
Instrumental Attitude									
MVPA support	5.14 (1.19)	5.65 (1.10)	5.81 (0.93)	5.92 (0.98)	6.05 (0.85)	6.17 (0.89)	26.55	0.11	1 < ALL; 2 < 5,6; 3 < 6
LPA support	5.08 (1.17)	5.62 (1.00)	5.81 (0.88)	5.95 (0.90)	6.09 (0.87)	6.23 (0.86)	36.49	0.15	1 < ALL; 2 < 5,6; 3 < 6
Sleep support	5.10 (1.23)	6.04 (0.99)	6.21 (0.86)	6.21 (0.92)	6.41 (0.78)	6.30 (0.89)	50.77	0.20	1 < ALL; 2 < 5
Screen support	4.51 (1.32)	4.89 (1.34)	5.88 (1.10)	5.29 (1.32)	6.06 (1.01)	6.05 (0.99)	41.20	0.22	1 < ALL; 2 < 3; 2,4 < 6,5
Perceived Capability									
MVPA support	3.72 (0.93)	3.88 (0.91)	4.06 (0.76)	4.16 (0.64)	4.28 (0.66)	4.42 (0.62)	21.01	0.09	1,2 < 5,6; 1 < 4; 3 < 6
LPA support	3.67 (0.88)	4.05 (0.81)	4.14 (0.60)	4.41 (0.53)	4.48 (0.55)	4.47 (0.59)	39.50	0.16	1 < ALL; 2 < 4,5,6; 3 < 5,6
Sleep support	3.58 (0.91)	4.31 (0.68)	4.38 (0.61)	4.43 (0.71)	4.61 (0.54)	4.45 (0.61)	57.45	0.22	1 < ALL; 2,3 < 5
Screen support	3.00 (1.06)	3.37 (1.04)	4.09 (0.82)	3.76 (0.99)	4.29 (0.73)	4.39 (0.62)	75.32	0.27	1 < 2 < ALL; 3 < 6; 4 < 5,6
Perceived Opportunity									
MVPA support	3.61 (1.00)	3.78 (0.99)	4.02 (0.83)	3.96 (0.91)	4.20 (0.93)	4.37 (0.68)	19.51	0.09	1,2 < 5,6; 1 < 3,4; 3,4 < 6
LPA support	3.50 (0.95)	3.91 (0.90)	4.03 (0.68)	4.32 (0.61)	4.44 (0.58)	4.44 (0.60)	45.37	0.18	1 < ALL; 2 < 4,5,6; 3 < 5,6
Sleep support	3.56 (0.95)	4.30 (0.75)	4.44 (0.61)	4.49 (0.65)	4.58 (0.59)	4.50 (0.62)	58.05	0.22	1 < ALL; 2 < 5
Screen support	2.94 (1.07)	3.36 (1.06)	4.10 (0.81)	3.78 (1.03)	4.34 (0.69)	4.35 (0.71)	78.81	0.29	1 < 2 < ALL; 4 < 5,6

Note: All analysis of variance results *p* < 0.01. *MVPA* Moderate to vigorous physical activity, *LPA* Light physical activity. Post Hoc tests are Bonferoni

Further analyses of the post-hoc tests of the associations between the social cognitive constructs and the six parental support clusters are provided in Table 3. In this table, the evidence for the predictive compatibility and generality of these constructs was compiled to shed further light on the results of Table 2. The results clearly show that compatibility associations were more prevalent than generality associations among the post hoc tests for MVPA (compatibility = 75% of tests; generality = 47% of tests), LPA (compatibility = 81% of tests; generality = 38% of tests), screen time (compatibility = 92% of tests; generality = 46% of tests), and sleep (compatibility = 100% of tests; generality = 22% of tests) support cognitions.

**Table 3:** Follow-up analyses of the specificity and generality associations of parental support social cognitions and regularly supporting their child in the behavioral clusters of the 24-Hour Movement Guidelines

	Specificity	Generality
MVPA Support		
Affective attitude	5/5 tests	5/9 tests
Instrumental attitude	3/5 tests	5/9 tests
Perceived Capability	3/5 tests	3/9 tests
Perceived Opportunity	4/5 test	4/9 tests
LPA Support		
Affective attitude	7/9 tests	3/6 tests
Instrumental attitude	6/9 tests	2/6 tests
Perceived Capability	8/9 tests	2/6 tests
Perceived Opportunity	8/9 tests	2/6 tests
Sleep Support		
Affective attitude	1/1 test	4/9 tests
Instrumental attitude	1/1 test	1/9 tests
Perceived Capability	1/1 test	2/9 tests
Perceived Opportunity	1/1 test	1/9 tests
Screen Restriction Support		
Affective attitude	9/9 tests	2/6 tests
Instrumental attitude	8/9 tests	2/6 tests
Perceived Capability	8/9 tests	4/6 tests
Perceived Opportunity	8/9 tests	3/6 tests

Note: Specificity is that the association between the social cognition for a specific behavior and its subsequent association with meeting the guideline for that behavior. Generality is the association between a social cognition for a behavior and its association with meeting the guideline for a different behavior or behaviors. *MVPA* Moderate to vigorous intensity physical activity, *LPA* Light intensity physical activity

## Discussion

The purpose of this paper was to explore the prevalence of parental support for meeting the 24-Hour Movement Guidelines and its component parts [7] among a representative sample of Canadian parents, identify key integrative support profiles among the four movement behaviors, and investigate subsequent sociodemographic and social cognitive correlates of these profiles. We hypothesized that sleep behavior was likely to have the highest prevalence of support among parents. This hypothesis was supported. Specifically, sleep (9–11 h for 5–12 year-olds) had the highest support with 72.7% of parents reporting they supported this behavior on most days of the week. The findings align with prior rationale that the benefits of parental support of sleep are well-established [31]. We also speculated that parental support of sleep behavior is likely higher than other movement behaviors because it is low burden (e.g., short in duration each night) and thus easier to accomplish, and that parents are most likely available for the opportunity to provide sleep support (e.g., home in the evening). There was indirect support for this hypothesis because screen time restriction (49.2% of parents supported at guidelines) and LPA (44.4% of parents supported at guidelines) followed by MVPA (23.2% of parents supported at guidelines) were all markedly lower than support for child and youth sleep. Physical activity is likely the most time-, resource-, and effort involved support behavior, with parental supports that span both encouragement and logistical elements. These results suggest that more intervention attention to parental support should be placed on MVPA, followed by LPA and screen-time, compared to sleep behavior.

Of course, the purpose of the Canadian 24-Hour Movement Guidelines for Children and Youth is to highlight the integration of sleep, LPA, MVPA, and sedentary behavior on health outcomes [7]. Therefore, the novel contribution of this research was to explore the interactive profiles of parental support across these four health behaviors. We had no specific a priori hypotheses about which of the 16 profiles would emerge to describe parents, but we did not expect these profiles to be equally distributed. This expectation was supported as only six of 16 potential support clusters had a meaningful sample size. These profiles were: 1. no support for any guideline (19.0%), 2. sleep support only (15.6%), 3. screen time and sleep support (15.1%), 4. LPA, screen time and sleep support (14.4%), 5. LPA and sleep support (6.8%), and 6. support of all guidelines (14.2%).

Several interesting findings emerged from these clusters. First, there was strong representation of parents at the poles of these support behaviors. A large proportion (~ a third) of the sample reported either providing no support for their child/youth at the level of the 24-Hour Movement Guidelines or provided full support of these behaviors at this level. This suggests considerable heterogeneity on the spectrum of parental support. Furthermore, the largest prevalence among all parent groups was for no support of the guidelines. This is concerning and highlights the importance of interventions. A second point of interest in these distributions was that sleep support behavior emerged as stand alone from all other health behaviors. While this echoes the very high prevalence of parents who support their child or youth’s sleep, it also highlights that sleep is not integrative as suggested by the premise of the 24-Hour Movement Guidelines. Finally, it was interesting to note that MVPA support was only featured in the cluster where all other guidelines were supported. This finding is commensurate with our rationale that MVPA support is likely the most burdensome behavior with considerable volume and scope of supports that are required [20, 23, 26, 28, 29]. Specifically, only the most involved parents, who supported their children at the level of all health behavior guidelines, also supported MVPA for their children and youth most days of the week.

Though five of these profiles of parental support highlight potential targets for intervention, an understanding of their correlates is required in order to develop effective interventions. Thus, the third purpose of our study was to explore the sociodemographic and social cognitive correlates of these parental support profiles of the Canadian 24-Hour Movement Guidelines for Children and Youth. For sociodemographic factors, we hypothesized that parents of younger children would be present in the higher support clusters (i.e., support of more health behaviors) than the lower support clusters compared to parents of adolescents. This hypothesis was supported. Indeed, a near linear relationship was observed between the number of health behaviors supported and the age of the child. Thus, the group who did not support at the level of the guidelines was comprised primarily of parents of teenagers. Some of the rationale for this age differentiation may be due to the autonomy afforded to developing youth by parents and thus a sensible approach to parenting [56]. Nevertheless, given the low prevalence of the 24-Hour Movement Guidelines among adolescents, this hands-off parenting approach may be premature and thus represents an important avenue for intervention upon parental support. This is somewhat counter-intuitive with the present research and intervention approaches that has a heavy focus on health behavior parenting in young children [57]. These data suggest that interventions for parental support among teenagers may be critical given the considerable opportunity for behavior to be shaped in positive ways that may improve longer term health outcomes [58].

The only other sociodemographic variable that was a consistent correlate across the support clusters was the gender of the parent. Fathers were associated with lower support of the 24-Hour Movement Guidelines compared to mothers. Though it has not been a consistent demographic correlate in any of these health behaviors in univariate analyses [15, 24, 25, 32–36, 59], these results suggest that the differences in father and mother support could be amplified through this examination of multiple health behavior support profiles. Further, the lack of engagement of fathers in health behaviors such as physical activity has been identified in past research even if this finding is not always consistent [2, 59–61] and a paucity of interventions focused on sedentary behavior and physical activity with a specific focus on fathers has been duly noted [62]. Our results are in agreement with this past research and supports focused interventions that target fathers in promoting the 24-Hour Movement Guidelines for Children and Youth.

To explore the social cognitive correlates of these interactive profiles of parental support, we employed key antecedent constructs of the TPB [37]. We hypothesized that higher values of all TPB constructs would be associated with higher support clusters (i.e., support of more behaviors) than lower support clusters compared to parents with lower values of these TPB constructs. The hypothesis was supported. The results are aligned with past research that has employed the TPB to understand [21, 25] and promote parental support of physical activity [22, 40, 41], yet these findings show that the TPB can also distinguish across health behavior support clusters of the 24-Hour Movement behaviors. There were some deviations in the predictive utility of the TPB depending on what health behavior was the focus of the questions. Specifically, social cognitions about MVPA support were consistently within the medium effect size range [55], LPA support and sleep cognitions were consistently in the low-end of the large effect size range, while screen support social cognitions were the highest reported effect sizes.

We also sought to explore the predictive compatibility of parental support social cognitions compared to their generality of prediction across other health behaviors in these interactive profiles. We hypothesized, based on TPB [37], that social cognitions about parental support would favor prediction compatibility over prediction generality. This hypothesis was supported. TPB constructs were able to predict their target behavior across 84% of the tests, while they predicted another health behavior in only 38% of tests. The results underscore that cognitions about support for one health behavior are not likely related to another health behavior. Thus, parents do likely require interventions that target each specific behavior where there is a deficit in support. While there is elegance in multiple behavior change interventions [43–45], these results generally support the premise that coordinated effort is needed with considerations of the unique features of each health behavior [63]. Nevertheless, assessment of social cognitions toward the overall integrated guidelines may better emphasize their interactivity and complementarity and future research would be helpful in order to test this possibility.

Despite the original findings of this paper, the results should be considered within the context of several limitations. First, the study features a passive cross-sectional design. Causal attributions in these types of designs are not possible. Second, our TPB model omitted the subjective norm construct and an examination of how norms as well as other potentially important correlates (habits, environmental cues) may relate to parental support seems useful in future research. Third, though dichotomizing the parental support variables was helpful in providing an understanding of these health behaviors at the threshold of the Canadian 24-Hour Movement Guidelines for Children and Youth, it is important to note that parents below these guidelines does not equate to “no support”. Fourth, our measures of parental support have shown predictive validity in past research [25, 42, 46, 47], but the validity of this measure has not been compared to other available measures of parental support. Finally, our sample showed generally strong representation of the Canadian population, but may not generalize to specific geographical locales or cultures. Future research is needed to test the generalizability of these findings.

## Conclusions

In summary, parental support has been established as an important variable linked to child and youth health behaviors. As parental support is a behavior unto itself, it is useful to understand its antecedents to inform future interventions. In this study, we explored the prevalence of parental support for meeting the 24-Hour Movement Guidelines for Children and Youth among a representative sample of Canadian parents, to identify key interactive support profiles among the four movement behaviors, and investigate subsequent sociodemographic and social cognitive correlates of these profiles. Our findings showed that child and youth sleep behavior had the highest parental support by a wide margin and MVPA support had the lowest prevalence. Interactive profiles of the four movement behaviors at the level recommended by the 24-Hour Movement Guidelines for Children and Youth yielded six primary clusters and comprised wide variation from parents who supported none of these behaviors, to parents who supported all four behaviors at the level recommended in these guidelines. These profiles could be distinguished by the age of the child (younger children had higher support) and the gender of the parent (mothers provided more support), suggesting some key targets for intervention. All profiles could be distinguished by constructs of the TPB (higher values corresponded with higher support), but TPB cognitions were more specific predictors of each health behavior rather than general predictors of aggregate health behavior clusters. The results provide important information on what content interventions may need to comprise (attitude, perceived behavioral control) to change parental support of child and youth movement behaviors while considering the unique features of each health behavior to maximize related intervention effectiveness.

## Supplementary information

**Additional file 1:** Definitions of Support Behaviors

**Additional file 2:** Measures of Health Behaviors

## Data Availability

All data is available from the first author upon request.
