# Supplementary material for: Parental support of the Canadian 24-hour movement guidelines for children and youth: prevalence and correlates
Source: BMC Public Health. 2019 Oct 28;19:1385. doi: 10.1186/s12889-019-7744-7 (PMC6816147; doi:10.1186/s12889-019-7744-7)
Supplement: Supplementary file 1 — Additional file 1: Definitions of Support Behaviors [file 12889_2019_7744_MOESM1_ESM.docx]

Table S1.

Definitions of Support Behaviors

| Health Behavior | Definition in Questionnaire |
| --- | --- |
| Moderate and vigorous intensity physical activity | Engaging in at least 60 minutes a day of activities that take physical effort and required the child to breath more than normal (e.g., hiking, skateboarding, cycling, running) [[1](#_ENREF_1)]. Parental support of their child’s MVPA was defined as encouraging their child’s participation in sport and physical activity on a regular basis; driving or supporting transportation for your child to engage in sport and physical activity; and/or playing sport or engaging in physical activity with your child [[2](#_ENREF_2), [3](#_ENREF_3)]. |
| Light intensity physical activity | Engaging in at least several hours of activities that do not result in sweat production or shortness of breath (e.g., mild stretching, playing with animals, walking) each day [[1](#_ENREF_1)]. We defined parental support their child’s LPA as encouraging their child to move around more; encouraging games, chores and activities that require movement; and going for walks or doing activities with their child [[2](#_ENREF_2), [3](#_ENREF_3)]. |
| Sleep | Engaging in uninterrupted 9 to 11 hours of sleep per night for those aged 5–13 years and 8 to 10 hours per night for those aged 14–17 years, with consistent bed and wake-up times [[1](#_ENREF_1)]. We defined parental support of their child’s sleep as encouraging their child’s bed time and wake-up times are on a regular basis, and ensuring that their child has the right setting for sleep (e.g., no TV, phone use at bed time or in the bedroom) [[4](#_ENREF_4)]. |
| Sedentary Screen Time | Engaging in no more than 2 hours a day of recreational screen time while sedentary [[1](#_ENREF_1)]. We defined parental restriction of their child’s screen time as encouraging their child to view screens no more than two hours a day while sedentary and ensuring that their child had other activities to pursue throughout the day that do not involve sitting (or lying down) and watching screens [[5](#_ENREF_5)]. |

**References**

1. Tremblay MS, Carson V, Chaput J-P, Connor Gorber S, Dinh T, Duggan M: **Canadian 24-hour movement guidelines for children and youth: An integration of physical activity, sedentary behaviour, and sleep**. *Applied Physiology, Nutrition and Metabolism* 2016, **41**:S311-327.

2. Rhodes RE, Spence JC, Berry T, Deshpande S, Faulkner G, Latimer-Cheung A, O’Reilly N, Tremblay M: **Understanding action control of parent support behavior for child physical activity**. *Health Psychology* 2016, **35**:131-140.

3. Rhodes RE, Spence JC, Berry T, Deshpande S, Faulkner G, Latimer-Cheung AE, O'Reilly N, Tremblay MS: **Predicting changes across 12 months in three types of parental support behaviors and mothers' perceptions of child physical activity**. *Annals of Behavioral Medicine* 2015, **49**:853-864.

4. Pyper E, Harrington D, Manson H: **Do parents’ support behaviours predict whether or not their children get sufficient sleep? A cross-sectional study**. *BMC Public Health* 2017, **17**:432.

5. Pyper E, Harrington D, Manson H: **The impact of different types of parental support behaviours on child physical activity, healthy eating, and screen time: a cross-sectional study**. *BMC Public Health* 2016, **16**:568.
