# Supplementary material for: Parental support of the Canadian 24-hour movement guidelines for children and youth: prevalence and correlates
Source: BMC Public Health. 2019 Oct 28;19:1385. doi: 10.1186/s12889-019-7744-7 (PMC6816147; doi:10.1186/s12889-019-7744-7)
Supplement: Supplementary file 2 — Additional file 2: Measures of Health Behaviors [file 12889_2019_7744_MOESM2_ESM.docx]

Table S2.

Measures of Health Behaviors

| Health Behavior | Question | Reference |
| --- | --- | --- |
| Moderate and vigorous intensity physical activity | “In a typical week, on how many days does your child engage in moderate-to-vigorous physical active for a total of at least 60 minutes per day?” The response format allowed parents to answer from zero to 7 days. | Adapted from Behavioral Risk Factor Surveillance Survey Instrument [[1](#_ENREF_1)], Health Behavior of School Aged Children Survey [[2](#_ENREF_2)], LSI index of the Godin Leisure-Time Exercise Questionnaire [[3](#_ENREF_3), [4](#_ENREF_4)], and the International Physical Activity Questionnaire [[5](#_ENREF_5)] |
| Light intensity physical activity | “In a typical week, on how many days does your child engage in light physical activity for several hours per day?” with a zero to 7 days response format. | Adapted from Behavioral Risk Factor Surveillance Survey Instrument [[1](#_ENREF_1)], Health Behavior of School Aged Children Survey [[2](#_ENREF_2)], LSI index of the Godin Leisure-Time Exercise Questionnaire [[3](#_ENREF_3), [4](#_ENREF_4)], and the International Physical Activity Questionnaire [[5](#_ENREF_5)] |
| Sleep | Child sleep used the item “How many hours does your child usually spend sleeping in a 24-hour period (including naps but excluding time spent resting)?” on a scale with response options from zero to 24 hours. | Adapted from the single item used to assess sleep duration in the Pittsburgh Sleep Quality Index [[6](#_ENREF_6)]. |
| Sedentary Screen Time | On average, how many total hours and minutes per day does your child watch TV, use the computer, and play video games, during their free time?” with a scale between 0-23 hours and 0-59 min. | Item used in the Canadian Health Measures Survey [[7](#_ENREF_7)]. |

**References**

1. CDC: **Behavioral risk factor surveillance system survey questionnaire**. Atlanta. GA: U.S. Department of Health and Human Services; 2001.

2. Janssen I, Katzmarzyk PT, Boyce WF, Vereecken C, Mulvihill C, Roberts C, Currie C, Pickett W: **Comparison of overweight and obesity prevalence in school-aged youth from 34 countries and their relationships with physical activity and dietary patterns**. *Obesity Reviews* 2005, **6**:123-132.

3. Godin G, Jobin J, Bouillon J: **Assessment of leisure time exercise behavior by self-report: A concurrent validity study.** *Canadian Journal of Public Health* 1986, **77**:359-361.

4. Godin G, Shephard RJ: **A simple method to assess exercise behavior in the community**. *Canadian Journal of Applied Sport Science* 1985, **10**:141-146.

5. Craig CL, Marshall AL, Sjostrom M, Bauman A, Booth M, Ainsworth B, Pratt M, Ekelund U, Yngve U, Sallis JF *et al*: **International physical activity questionnaire: 12-country reliability and validity**. *Medicine and Science in Sports and Exercise* 2003, **35**(8):1381-1395.

6. Buysse DJ, Reynolds CF, Monk TH, Berman SR, Kupfer DJ: **The Pittsburgh Sleep Quality Index (PSQI): A new instrument for psychiatric research and practice**. *Psychiatry Research* 1989, **28**(2):193-213.

7. Statistics Canada: **Canadian Health Measures Survey Household Questionnaire**. In*.* Ottawa, ON: Statistics Canada; 2015.
